# Evaluating the effect of birth weight on brain volumes and depression: An observational and genetic study using UK Biobank cohort

**DOI:** 10.1192/j.eurpsy.2020.74

**Published:** 2020-07-24

**Authors:** Jing Ye, Cuiyan Wu, Xiaomeng Chu, Yan Wen, Ping Li, Bolun Cheng, Shiqiang Cheng, Li Liu, Lu Zhang, Mei Ma, Xin Qi, Chujun Liang, Om Prakash Kafle, Yumeng Jia, Sen Wang, Xi Wang, Yujie Ning, Feng Zhang

**Affiliations:** 1 Key Laboratory of Trace Elements and Endemic Diseases of National Health and Family Planning Commission, School of Public Health, Health Science Center, Xi’an Jiaotong University, Xi’an, China

**Keywords:** Birth weight, brain volume, depression, polygenic score

## Abstract

**Background.:**

Birth weight influences not only brain development, but also mental health outcomes, including depression, but the underlying mechanism is unclear.

**Methods.:**

The phenotypic data of 12,872–91,009 participants (59.18–63.38% women) from UK Biobank were included to test the associations between the birth weight, depression, and brain volumes through the linear and logistic regression models. As birth weight is highly heritable, the polygenic risk scores (PRSs) of birth weight were calculated from the UK Biobank cohort (154,539 participants, 56.90% women) to estimate the effect of birth weight-related genetic variation on the development of depression and brain volumes. Finally, the mediation analyses of step approach and mediation analysis were used to estimate the role of brain volumes in the association between birth weight and depression. All analyses were conducted sex stratified to assess sex-specific role in the associations.

**Result.:**

We observed associations between birth weight and depression (odds ratio [OR] = 0.968, 95% confidence interval [CI] = 0.957–0.979, *p* = 2.29 × 10^−6^). Positive associations were observed between birth weight and brain volumes, such as gray matter (*B* = 0.131, *p* = 3.51 × 10^−74^) and white matter (*B* = 0.129, *p* = 1.67 × 10^−74^). Depression was also associated with brain volume, such as left thalamus (OR = 0.891, 95% CI = 0.850–0.933, *p* = 4.46 × 10^−5^) and right thalamus (OR = 0.884, 95% CI = 0.841–0.928, *p* = 2.67 × 10^−5^). Additionally, significant mediation effects of brain volume were found for the associations between birth weight and depression through steps approach and mediation analysis, such as gray matter (*B* = –0.220, *p* = 0.020) and right thalamus (*B* = –0.207, *p* = 0.014).

**Conclusions.:**

Our results showed the associations among birth weight, depression, and brain volumes, and the mediation effect of brain volumes also provide evidence for the sex-specific of associations.

## Introduction

Barker’s hypothesis suggested that early in development, especially during life in the intrauterine life, adverse effects can lead to permanent changes in physiology and metabolism, resulting in an increased risk of disease in adulthood [1]. Birth weight is considered to be an important predictor of neonatal and infant survival and is associated with the risk of noncommunicable, chronic illnesses in the offspring [2], especially metabolic diseases [3, 4] and cardiovascular diseases [3,4]. The development of brain can also be influenced by birth weight, which might lead to the unhealthily physical and mental development, even psychological illness [5–8]. For instance, some literature reported that birth weight was associated with adult cortical surface area hippocampal volume, which was mainly responsible for long-period memory storage conversion and orientation [5,6]. Wald et al. indicated the effects of birth weight variation within the normal range on children’s neuropsychological function, including language, executive functioning, and theory of mind [7].

Previous studies also observed relationship between birth weight and depression. Warrington et al. conducted a study about maternal and fetal genetic effects on birth weight, and demonstrated genetic correlation between birth weight and mental disorder through linkage disequilibrium (LD) score regression, such as major depressive disorder and bipolar disorder (BD) [4]. However, previous literature showed that the association between depression and birth weight remains controversial [9–12]. For example, an epidemiological study did not find compelling association between low birth weight and suspected major depression from early adolescence to early adulthood [11]. Another systematic review and meta-analysis displayed a weak association between low birth weight and later depression or psychological distress [12].

Genetic factors contribute greatly to the variations of birth weight, brain volume and depression. Resent genome-wide association studies (GWAS) meta-analyses of own birth weight identified 146 independent single-nucleotide polymorphisms (SNPs) at genome-wide significance [4]. Zhao et al. performed a GWAS on 101 brain volume phenotypes using UK Biobank including 19,629 participants, identifying 365 independent genetic variations [13]. Twin and family estimates suggest that approximately 30–40% of the unipolar depression can be explained by genetic effects [14]. Clarifying the genetic relationships between birth weight, depression and brain volumes can provide important clues for understanding the potential impact of birth weight on brain volumes and depression.

Polygenic risk score (PRS) analysis is a powerful approach that aggregates the effects of genetic variants from GWAS summary data to identify the relationship between traits and diseases. From the Suffolk County Mental Project, an inception cohort study of first-admission patients with psychosis, Jonas et al. indicated that schizophrenia (SZ) PRS was the strongest predictors of diagnostic shifts from affective to nonaffective psychosis and could predict whether patients who appear to have a mood disorder with psychotic at first admission will eventually be diagnosed with a SZ spectrum disorder [15]. Another PRS study provided evidence that BD with manic psychosis genetically was more similar to SZ than any other tested BD subgroup through comparing SZ PRSs of different psychosis subtypes of BD [16].

Utilizing the UK Biobank cohort, we first tested the associations between birth weight, depression, and brain volumes. The PRSs of birth weight were then calculated from the individual-level genotype data of UK Biobank cohort. Linear and logistic regression models were then performed to evaluate the genetic impact of birth weight on depression and brain volume. Finally, the mediation model was used to estimate the role of brain volume in the association between birth weight and depression. All analyses were conducted sex stratified to assess sex-specific role in the associations.

## Aims of the Study

This study attempted to explore: (a) associations between birth weight on brain volumes; (b) associations between brain volumes on depression; and (c) associations between birth weight on depression, and whether brain volumes plays a mediating role in it.

## Methods

### UK Biobank cohort

Study individuals were from the UK Biobank health resource, which has performed a large prospective population-based cohort study, comprising linked health, hospital-record and genetic data of 502,656 participants aged 40–69 years in 2006 and 2010. All participants agreed to use their anonymous data and samples for any health-related research, to reconnect for further substudies [17]. The present study was conducted under UK Biobank approvals, to access participants’ health-related records, including birth weight from screenshot question or verbal interview within Assessment Center, self-reported depression status and brain volume from magnetic resonance imaging assessments.

### UK Biobank phenotypes of birth weight, depression, and brain

Participants were asked to enter their own birth-weight through verbal interview with 277,000 participants completed (UK Biobank field ID: 20022). In order to obtain a comprehensive and accurate control group of depressed and nondepressed patients, Patient Health Questionnaire (PHQ-9) and another strict criterion based on composite international diagnostic interview short-form (CIDI-SF) [18,19] were used to define the inclusion and exclusion criteria (full details in Appendix in the Supplementary Material). The following indicators, volume of gray matter (UK Biobank code: 25006), white matter (UK Biobank code: 25008), gray and white matter (UK Biobank code: 25010), left and right thalamus (UK Biobank code: 25011:25012), left and right accumbens (UK Biobank code: 25023:25024), gray matter in left frontal pole and gray matter in right frontal pole (UK Biobank code: 25782:25783), and gray matter in left VIIIa cerebellum and gray matter in right VIIIa cerebellum (UK Biobank code: 25909 and 25911) were selected as the measurement of brain structure. The total intracranial volume was defined as the sum of total gray matter, white matter, and cerebrospinal fluid. Birth weight and brain volume were mean-centered and normalized to one standard deviation (SD) before further analysis.

### UK Biobank genotyping, imputation, and quality control

A total of 488,377 participants were genotyped undertaking either the UK Bileve array or the UK Biobank axiom array under favorable quality control. Details of the array design, genotyping, and quality control procedures have been described elsewhere [20]. These genotypes were subsequently imputed to the Haplotype Reference Consortium (HRC) reference panel [21] (version 1.1) and UK10K and 1000 Genomes project reference panels [20]. We applied additional quality control filters to select high-quality SNPs: SNPs with high linkage disequilibrium (*r* ^2^ > 0.5) were pruned. We removed the participants, who reported inconsistencies between self-reported gender and genetic gender, who were genotyped but not imputed, and who withdraw their consents. Additionally, individuals were restricted to only “white British” based on self-reported ethnicity (UK Biobank field ID: 21000). UK Biobank participants was used to obtain relatively independent SNPs through King software.

### GWAS datasets of birth weight and PRS calculation

The GWAS summary statistics of birth weight [3] was from a recent meta-analysis of early growth genetics (EGG) consortium, combining data from EGG consortium and the fully released data of UK Biobank (released May 2017). Briefly, data from EGG consortium were imputed against the 1000 Genomes Project (Phase 1 v3) reference panel and data from UK Biobank were imputed against the HRC reference panel. The association between each genetic variant and birth weight measure was tested using linear regression in EGG consortium and a linear mixed model in UK Biobank, with adjustment for gestational age. We selected the SNP with genome-wide association testing *p* value <5.0 × 10^−8^ to calculate PRSs for all individuals. The GWAS summary statistics of European-only meta-analysis of own birth weight including 298,142 individuals was used in the present study. More details about the GWAS of birth weight were described in the previous study [3].

The PRS of birth weight was calculated as the sum of allele, weighted by their effect sizes, defined by 
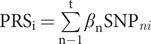
, where *n* (*n =* 1, 2, 3, …, *t*) and *i* (*i =* 1, 2, 3, …, *k*) denote the number of genetic markers and the sample size, respectively. *β_n_* represents the effect value of risk allele of the *n*th significant SNP related to birth weight obtained from the previous study [3]. SNP*_ni_* represents the risk allele dosage of the *n*th SNP of the *i*th individual. At last, 626 SNPs associated with birth weight driven from EGG consortium were used for PRS calculation by using PLINK’s“--score” command [22]. We conducted data centralization and standardization of the PRSs of birth weight.

### Statistical analysis

We applied a widely used approach for mediation analysis: the causal steps approach, which was described by Kenny et al. [23] (The detailed information of the causal steps approach was provided in the Supplementary Material). First, logistic regression analyses were conducted to estimate the associations between birth weight and depression, where birth weight phenotype or related PRS was used as an exposure variable, and depression was used as an outcome variable. Second, linear regression analyses were applied for evaluating the associations between birth weight and brain volumes, where birth weight phenotype or related PRS was used as exposure variable and brain volumes were used as outcome variables. Third, logistic regression analyses were conducted to estimate the associations between brain volumes and depression, where brain volumes were used as exposure variables, and depression was used as an outcome variable. Fourth, logistic regression analyses were also performed for depression and birth weight, where each of brain volumes was used as a covariate to explore the role of brain volume in the association between birth weight and depression. For each regression analysis, sex, age, and 10 principle components of population structure, Townsend deprivation and alcohol use were used as covariates, and total intracranial volume was only used as a covariate for the third and fourth steps. All analyses were conducted sex stratified to assess sex-specific role in the associations through R 3.5.3.

### Sensitive analyses

We used the R package “mediation,” which relied on the causal steps approach [23] for the mediation analysis, and conducted the similar analyses with the same data. We used the mediate function to estimate the indirect effect (or average causal mediation effect (ACME)) and direct effect (or average direct effect (ADE)) of birth weight to depression. The mediation model needs two statistical models, the one is the linear regression model of the exposure variable (birth weight) and mediate variable (brain volume), the other one is the logistic regression model of the exposure variable (birth weight), mediate variable (brain volume), and outcome variable (depression). The default number of simulation (1000) was used, which type is the quasi-Bayesian Monte Carlo method based on normal approximation [24]. From statistics level, only when the *p* value of ACME result was significant, suggesting that the brain volumes play the mediating role in the association between birth weight and depression. All analyses were conducted sex stratified to assess sex-specific role.

## Result

### Descriptive characteristics of study samples

Table 1 shows the descript characteristics of study samples.

**Table 1.** Characteristic of participants.

		Birth weight (PRS)	Birth weight (phenotype)
Depression	Case/control	75,674/7,8865	45,044/45,965
	Sex (female)	87,930 (56.90%)	57,684 (63.38%)
	Age (SD)	56.10 (7.77)	54.85 (7.79)
Volume of gray, white and gray + white matter	Sample	21,502	12,872
	Sex (female)	11,346 (52.77%)	7,618 (59.18%)
	Age (SD)	54.88 (7.49)	53.63 (7.41)
Volume of thalamus and accumbens	Sample	21,484	12,865
	Sex (female)	11340 (52.78%)	7,614 (59.18%)
	Age (SD)	54.88 (7.49)	53.63 (7.41)
Volume of gray matter in frontal pole and in VIIIa cerebellum	Sample	21,496	12,870
	Sex (female)	11,345 (52.78%)	7,617 (59.18%)
	Age (SD)	54.88 (7.49)	53.63 (7.41)

Age was described as mean (standard deviation [SD]).

Abbreviation: PRS, polygenic risk scores.

### Associations between birth weight and depression

We observed associations between birth weight and depression (odds ratio [OR] = 0.968, 95% confidence interval [CI] = 0.957–0.979, *p* = 2.29 × 10^−6^), as well as birth weight PRS and depression (OR = 0.984, 95% CI = 0.975–0.993, *p* = 0.002). In women, depression was associated with birth weight phenotype (OR = 0.962, 95% CI = 0.948–0.975, *p* = 4.87 × 10^−6^) and PRS (OR = 0.986, 95% CI = 0.974–0.997, *p* = 0.035). In men, depression was only associated with birth weight PRS (OR = 0.980, 95% CI = 0.961–0.998, *p* = 0.028) (Table 2).

**Table 2.** Association between depression and birth weight.

**Outcome**	**Exposure**	**Sex**	**B**	**P**	**OR (95%CI)**
Depression	Birth weight (phenotype)	All	−0.033	2.29 × 10^−06^	0.968(0.957–0.979)
Depression	Birth weight (PRS)	All	−0.016	0.002	0.984(0.975–0.993)
Depression	Birth weight (phenotype)	Men	−0.021	0.071	0.980(0.961–0.998)
Depression	Birth weight (PRS)	Men	−0.018	0.028	0.982(0.969–0.995)
Depression	Birth weight (phenotype)	Women	−0.039	4.87 × 10^−06^	0.962(0.948–0.975)
Depression	Birth weight (PRS)	Women	−0.015	0.035	0.986(0.974–0.997)

Logistic regression was used to test the association of depression and birth weight (PRS and phenotype). Birth weight PRS indicates the polygenic scores for birth weight. Birth weight phenotype means the phenotype of birth weight.

Abbreviation: PRS, polygenic risk scores.

### Associations between birth weight and brain volumes

**Figure 1.**
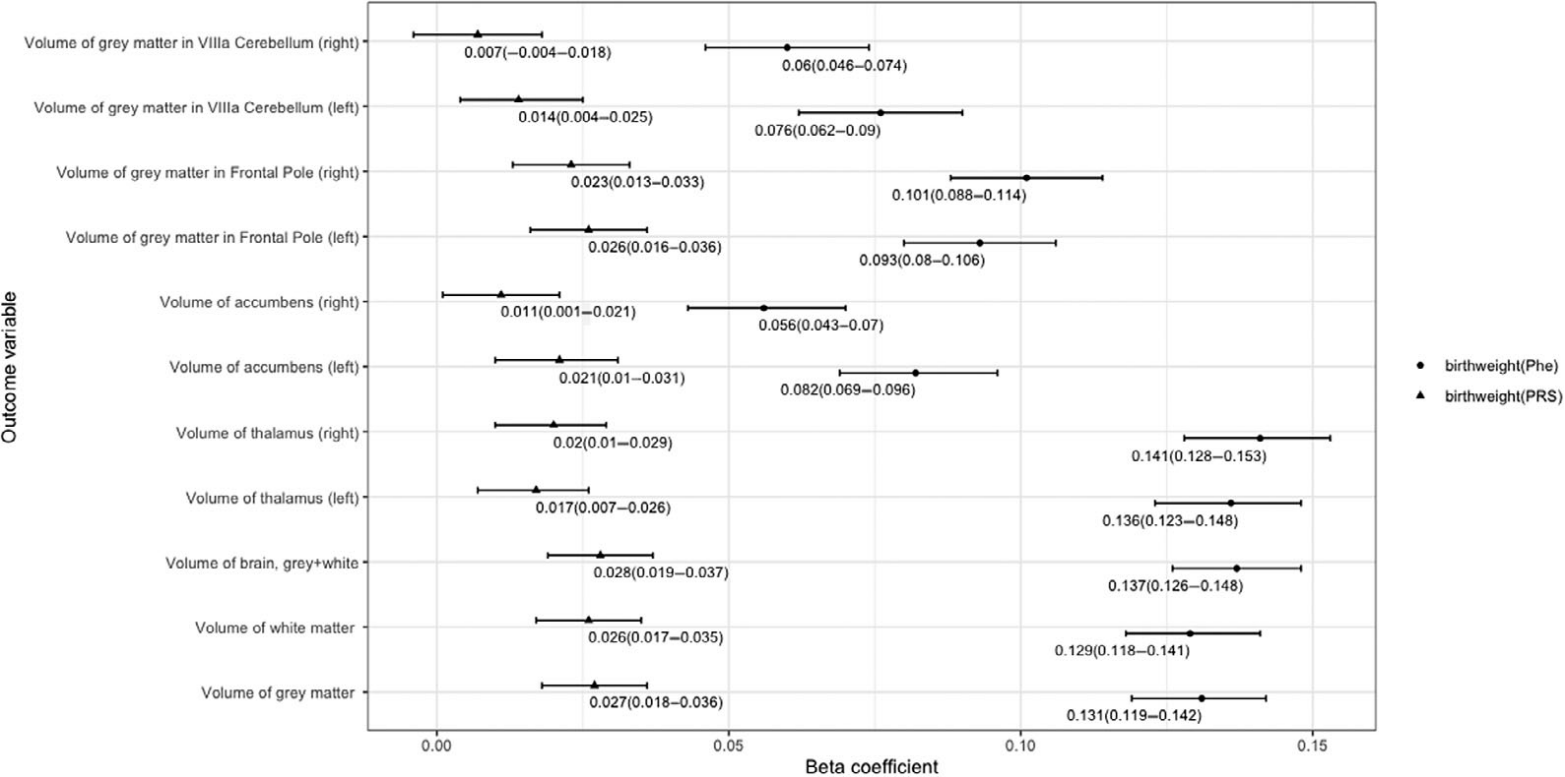
Associations between birth weight and brain volume. The *x*-axis refers to beta coefficient. The *y*-axis represents the outcome variables. Points display the beta and 95% confidence intervals (CIs) (error bars) of beta coefficient. Birth weight polygenic risk scores (PRS) indicates the polygenic scores for birth weight. Birth weight phenotype means the phenotype of birth weight. Detail information is showed in Table S2 in the Supplementary Material.

As shown in Figure 1, we observed the associations between birth weight phenotype and the brain volumes, such as gray matter (*B* = 0.131, *p* = 3.51 × 10^−74^), gray and white matter (*B* = 0.137, *p* = 5.70 × 10^−85^), and thalamus (*B* = 0.136, *p* = 3.09 × 10^−72^ for left and *B* = 0.141, *p* = 1.86 × 10^−79^ for right). More additional details were provided in Table S1 in the Supplementary Material. In men and women, we obtained the similar results that birth weight was significant associated with each of the brain volumes. Detailed information was provided in the Figure 3, Figures S2 and S3 and Tables S2 and S3 in the Supplementary Material.

As shown in Figure 1, further PRS analysis also observed genetic associations between birth weight and brain volumes, such as gray matter (*B* = 0.027, *p* = 8.80 × 10^−7^), gray and white matter (*B* = 0.028, *p* = 2.41 × 10^−7^), and thalamus (*B* = 0.017, *p* = 0.004 for left and *B* = 0.020, *p* = 5.86 × 10^−4^ for right). More additional details were provided in Table S1 in the Supplementary Material. In men, we obtained the similar results that birth weight PRS was significant associated with each of the brain volumes, except the volume of gray matter in VIIIa cerebellum. And in women, birth weight PRS was only associated with the brain volume of gray matter, white matter, gray and white matter, left accumbens, and gray matter in frontal pole. Detailed information was provided in the Figure 3 Figures S2 and S3 and Tables S2 and S3 in the Supplementary Material.

### Associations between depression and brain volumes

**Figure 2.**
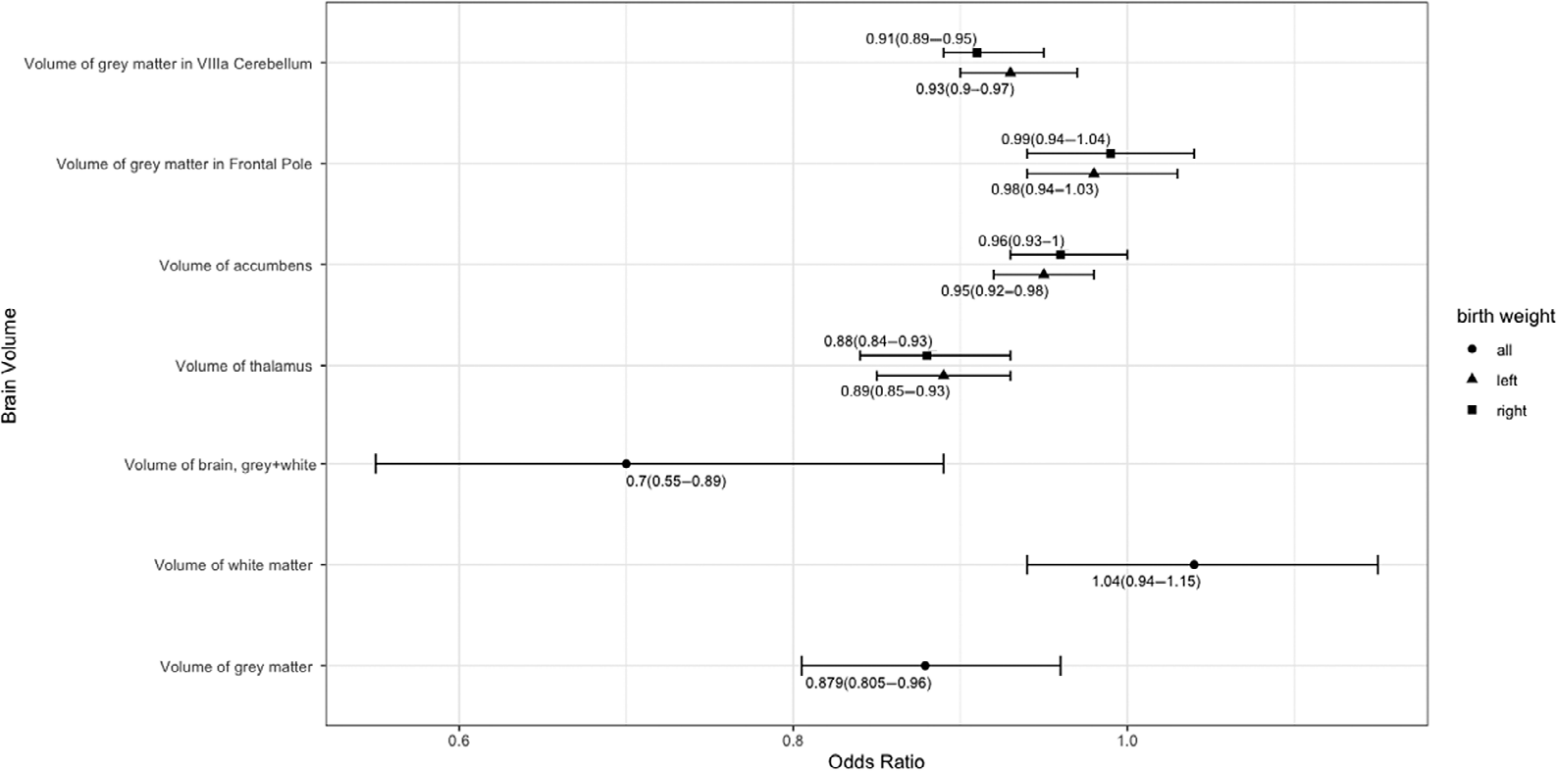
Association between depression and brain volume. The *x*-axis refers to odds ratio (OR). The *y*-axis represents the exposure variables. Points display the OR and 95% confidence intervals (CIs) (error bars) of OR. Detail information is showed in Table S3 in the Supplementary Material.

**Figure 3.**
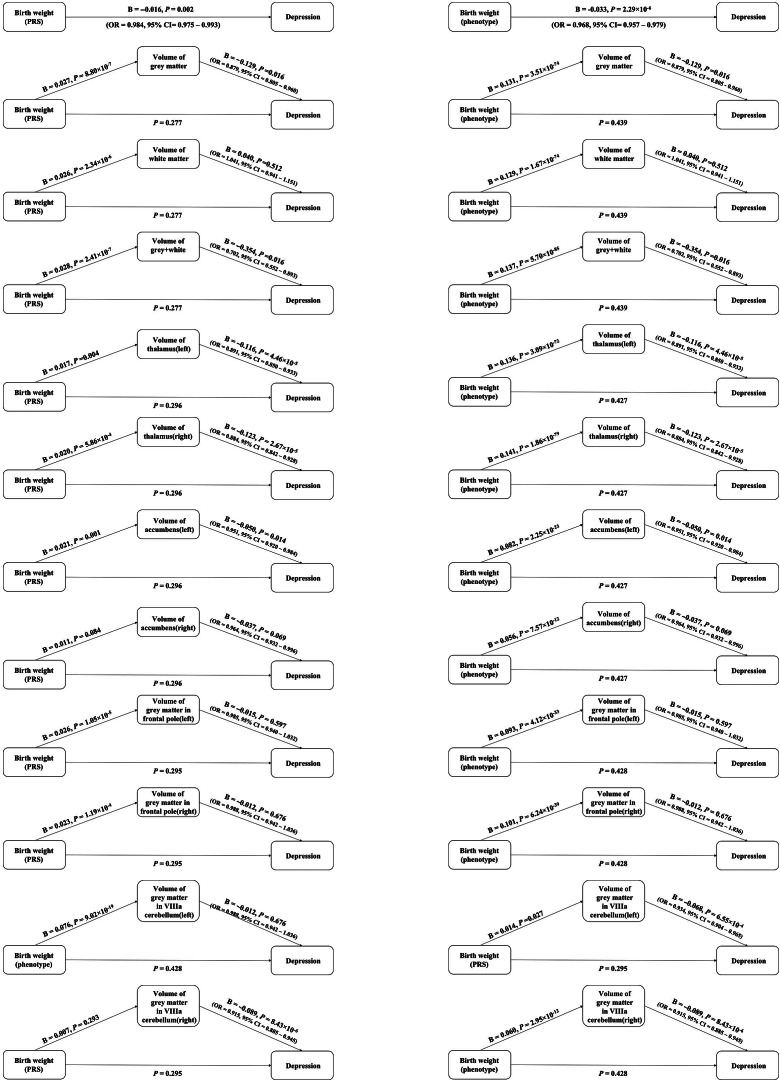
Association between birth weight and depression via elevated levels of one of brain volumes through steps approach. Birth weight polygenic risk scores (PRS) indicates the polygenic scores for birth weight. Birth weight phenotype means the phenotype of birth weight.

As shown in Figure 2, associations were observed between depression and brain volumes, such as gray matter (OR = 0.879, 95% CI = 0.805–0.960, *p* = 0.016), gray and white matter (OR = 0.702, 95% CI = 0.552–0.893, *p* = 0.016), left thalamus (OR = 0.891, 95% CI = 0.850–0.933, *p* = 4.46 × 10^−5^), and right thalamus (OR = 0.884, 95% CI = 0.841–0.928, *p* = 2.67 × 10^−5^). More additional details were provided in Table S4 in the Supplementary Material. In men, we only obtained that depression was significant associated with gray matter and gray matter in right VIIIa cerebellum. And in women, depression was associated with the brain volume of gray and white matter, thalamus, accumbens and gray matter in VIIIa cerebellum. Detailed information was provided in the Figure 3, Figures S4 and S5 and Tables S5 and S6 in the Supplementary Material.

### Associations between birth weight and depression adjusting for brain volume as covariate

We observed no significant association between birth weight (both phenotype and PRS) and depression, when one of the brain volumes was used as a covariate (all *p* value >0.05). After gender classified, we observed the same results. Detailed information was provided in the Figure 3, Figures S6 and S7 and Tables S7–S9 in the Supplementary Material.

### Summary of mediation effects of brain volumes between birth weight and depression

Through the causal steps approach, parts of the brain volumes were the complete mediators between birth weight and depression. For birth weight phenotype, the brain volumes included gray matter, gray and white matter, left thalamus, right thalamus, left accumbens, and gray matter in left VIIIa cerebellum. In men, gray matter and gray matter in left VIIIa cerebellum played the mediation effects between birth weight and depression. And in women, left and right thalamus, left and right accumbens and gray matter in left VIIIa cerebellum played the mediation effects between birth weight and depression.

For birth weight PRS, the brain volumes included the brain volumes included gray matter, gray and white matter, left and right thalamus, left accumbens, and gray matter in left VIIIa cerebellum. In men, only gray matter played the mediation effects between birth weight and depression. And in women, only left accumbens played the mediation effects between birth weight and depression. More additional details were provided in Figure 3 and Figures S6 and S7 in the Supplementary Material.

### Sensitive analyses

We obtained the similar results from mediation analyses through R package. The indirect path from birth weight PRS that were based on a GWAS to depression via elevated levels of one of the brain volumes, including gray matter, gray and white matter, left thalamus, right thalamus, and left accumbens. More additional details were provided in Tables S10–S12 in the Supplementary Material.

## Discussion

We conducted a large observational and genetic PRS study to evaluate the associations between birth weight, brain volume, and depression using UK Biobank population cohort. We observed phenotypic associations for birth weight versus brain volumes, birth weight versus depression, and depression versus brain volumes. Further PRS analysis also provided evidence for genetic predispositions between birth weight, depression and brain volumes. It is interesting that the correlation between birth weight and depression was not significant after adjusting the impact of brain volume, suggesting that brain volumes might be an important mediator between birth weight and depression.

Previous studies have demonstrated that birth weight was associated with brain development [25–27], which is consistent with our results. Our results indicated the positive association between birth weight and multiple brain volumes. A meta-analysis of birth weight concluded that birth weight was associated with the volume of white matter, gray matter, gray and white matter, and thalamus [28]. In this study, we also detected several novel brain regions to test the association with birth weight, such as accumbens volume and frontal pole volume. Additionally, limited literature provided evidence for the genetic association between birth weight and brain volume. For instance, rs8756 and rs7968682 were reported to be associated with brain structure [4]. Through a GWAS of regional brain volume in 19,629 individuals, authors indicated a significant correlation between depressive symptoms and gray matter [13]. Our PRS analysis results provided novel evidence for the effects of birth weight related genetic factors on the variations of brain volumes. For the reason why low birth weight is risk factor for brain development, combining with previous studies [29,30], one possible explanation is that low birth weight may affect normal brain development and maturation after birth, eventually leading to abnormal brain volumes in adults.

Meanwhile, association was also observed between brain volume and depression in this study. It has been suggested that decreased volume of the hippocampus, anterior cingulate cortex, prefrontal cortex, striatum and amygdala, and impaired white matter integrity are often observed in depressed adults [25]. Merz et al. conducted a significant correlation between higher levels of depressive symptoms and lower levels of hippocampal gray matter and larger volumes of pale gray matter in 328 children and adolescents between the ages of 7 and 21 [31]. In this study, we found that depression was associated with multiple brain volumes, such as gray matter, brain of gray and white matter, left and right thalamus, left and right accumbens, gray matter in left and right frontal pole, and VIIIa cerebellum. Interestingly, most of the brain volumes that were associated with birth weight were also associated with depression in this study.

As described in the introduction section, the association between birth weight and depression remains controversial [9–12]. Some of previous studies proved that low birth weight increased the risk of depressive disorder [9,10], which was consistent with the present study. However, Levine et al. demonstrated that there is a lack of convincing evidence to support an association between very low birth weight and suspected major depression, taking into account well-documented confounding factors [11]. Our results shown that not only the increase of the birth weight phenotype, but also the PRS for birth weight can lead to the decreased prevalence of depression, which also meant the genetic liability between birth weight and depression. Combined with the insignificant results of other studies [11,12], we may speculate that the effect of birth weight on depression was not direct but indirect.

Based on the results, higher birth weight was associated with higher brain volumes, higher brain volumes were associated with lower morbidity of depression, and the weak association between depression and birth weight, we inferred that the brain volumes might play an important role for the observed association between birth weight and depression. This hypothesis was supported by the fact that the association between birth weight and depression disappeared, after adjusting the one of brain volumes as a covariate in regression analysis and significant indirect path results from the mediation approach. The possible mechanism is that the low brain volume of low birth weight leads to neuropathologic changes in adulthood, resulting in the risk of depression. Also, birth weight may affect the risk of depression through other mediating variables, so that more researches are expected.

Using the individual-level phenotypic and genetic and data from the UK Biobank, the large sample size and a wide range of variables make our conclusions more rigorous and convincing. Using PRS method to explore the effect of birth weight on depression and brain volume is a powerful method to reduce the impact of nongenetic confounding factors on our study results.

There were some limitations to this study. First, depression was measured retrospectively by self-report through an online questionnaire and birth weight was collected from participants’ recall, which may increase the likelihood of measurement errors. The conclusions are mainly applicable to middle-aged and elderly European populations, lacking comparisons among the young and the interracial populations. Second, the study is a cross-sectional study. Therefore, a causal relationship between depression and brain volume cannot be established. Third, it could be at birth persons with low birth weight had different brain development at birth, through the brain dysplasia of low birth weight [29,30]. Although limited studies suggested that age had no effect on the difference in brain volume between low birth weight and normal weight [32,33], further longitudinal studies are expected to prove the continuity of mediation. At last, there are some other confounders might influence depression. Although we chose Townsend deprivation index and alcohol use as covariates according to the previous studies [34–36], some another possibly confounders were not considered in this study and need further research.

Due to treatment for depression can only alleviate rather than cure the condition, and the burden of depression outcome continues to grow. Therefore, the identification and prevention of the high-risk group of depression is an effective and key measure. Our study showed that birth weight was positively correlated with depression, negatively correlated with brain volumes and brain volume might an important mediator between birth weight and depression. Therefore, this study suggested that paying more attention to the birth weight and brain development might be helpful for early prevention and treatment of depression.

## Data Availability

The UKB data are available through the UK Biobank Access Management System https://www.ukbiobank.ac.uk/. We will return the derived data fields following UKB policy; in due course, they will be available through the UK Biobank Access Management system.

## Code Availability.

a<−lm (brain_volume~birth_weight+covariate).

b<−glm(depression~brain_volume+covariate,family=binomial).

c<−mediate(a,b,treat=‘birth_weight’, mediator=‘brain_volume’).

summary(c).

## Supplementary material

For supplementary material accompanying this paper visit http://dx.doi.org/10.1192/j.eurpsy.2020.74. click here to view supplementary material
