# Supplementary material for: Evaluating the effect of birth weight on brain volumes and depression: An observational and genetic study using UK Biobank cohort
Source: Eur Psychiatry. 2020 Jul 24;63(1):e73. doi: 10.1192/j.eurpsy.2020.74 (PMC7503174; doi:10.1192/j.eurpsy.2020.74)
Supplement: Supplementary file 1 [file S0924933820000747sup.zip › S0924933820000747sup001.docx]

**Supplement**

***Definitions of criterion for depression***

In order to obtain a comprehensive and accurate control group of depressed and non-depressed patients, we self-reported depression, and strictly conducted the control group threshold by Davis et al. research^1^, which based on Patient Health Questionnaire (PHQ-9)^2^ and another strict criterion based on composite international diagnostic interview short-form (CIDI-SF)^2,3^.

Depression phenotype was defined according to three UK Biobank fields: 20002, 20126 and 20544. We selected participants who self reported depression, based on the code 1286 from ID 20002, code 3,4 or 5 from ID 20126 and code 11 from ID 20544 as case.

And for the control of the depression, after excluding the self reported depression defined in our study and depression single episode defined in Davis et al. research^1^,we chose the participants who did not endorse depression or screen positive on PHQ^2^ or CIDI^3^. More precisely, participants whose PHQ score ≤5 and did not have core symptoms were selected.

PHQ-9 is a classification algorithm with a total score (0-27) used to screen for and measure depression severity, focusing on nine depressive symptoms and signs (as detailed below：Little interest or pleasure in doing things 20514, Feeling down, depressed, or hopeless 20510, Trouble sleeping 20517, Feeling tired 20519, Poor appetite or overeating 20511, Feeling bad about yourself 20507, Trouble concentrating 20508, Moving or speaking slowly or fidgety or restless 20518, Thoughts that you would be better off dead 20513). In order to meet the 0-3 score for each item of PHQ, the 9 symptom scores (1-4) of our team UK were all reduced by 1 point, which was then added up and participants with PHQ≤5 were selected.

According to CIDI, core symptoms of depression were ID 20446 and ID 20441 in UK Biobank, we chose the participants who response “NO” to the question “Have you ever had a time in your life when you felt sad, blue, or depressed for two weeks or more in a row?” or “Have you ever had a time in your life lasting two weeks or more when you lost interest in most things like hobbies, work, or activities that usually give you pleasure?” as non-depressed patients.

**Definitions of criterion for alcohol use**

There are several phenotype options that measure drinking behavior in the UKB. After considering only phenotypes that cover the entire UKB sample, we were left with one: ever alcohol drinking. We coded participant status as 1 if a respondent reported that they were drinking current or previous and 0.

**The causal steps approach: a mediation analysis**

The mediation effect test procedure described by Kenny and his colleagues4 is a test procedure with a lot of use. This method is easy to understand and operate. The specific steps are as follows (Figure 1):

1) Test whether the total effect coefficient c is significant, that is, whether there is a significant relationship between independent variables and dependent variables. If c is significant, the subsequent analysis continues, and if not, the mediation analysis terminates.

2) Test whether the effect of independent variable on the mediation variable a is significant; If a is significant, the subsequent test will be continued; otherwise, the analysis will be terminated and the mediating effect will not exist.

3) Test whether the effect of the mediation variable on the dependent variable is b significant; If b is significant, the subsequent test is continued; otherwise, the analysis is terminated and the mediating effect does not exist.

4) Test whether the direct effect c' is significant. In the case that both a and b are significant, if c' is not significant, there is a complete mediation5. Otherwise, there is a partial mediation effect4.

**
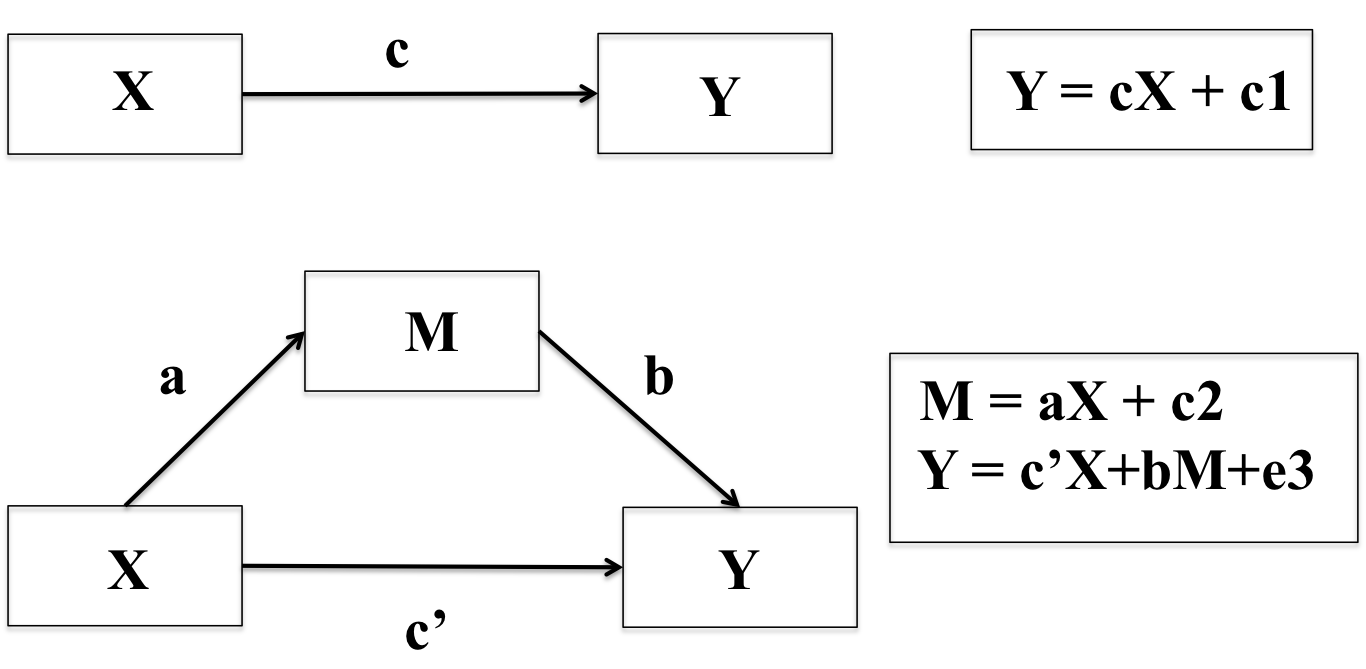
Figure S1. The flow chart of the causal steps approach**

* X is the instrumental variable; Y is the outcome variable; M is the mediate variable.

**Figure S2. Associations between birth weight and brain volume for men**


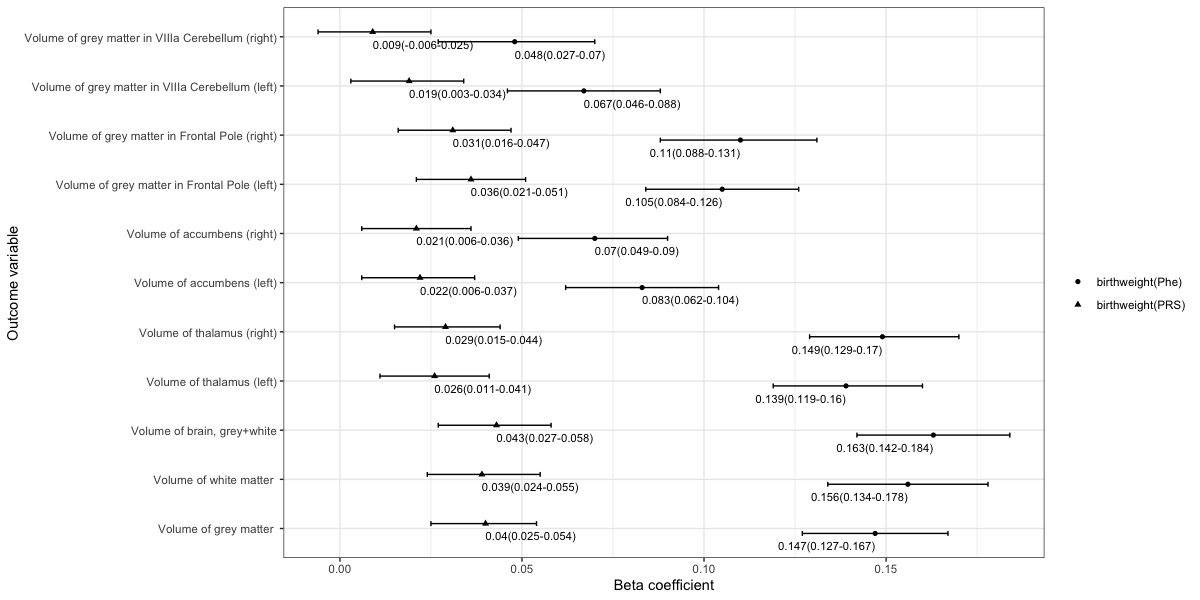


*The x-axis refers to beta coefficient. The y-axis represents the outcome variables. Points display the beta and 95% CIs (error bars) of beta coefficient. Birth weight PRS indicates the polygenic scores for birth weight. Birth weight Phenotype means the phenotype of birth weight. Detail information is showed in Table S2 in the Supplement.

**Figure S3. Associations between birth weight and brain volume for women**


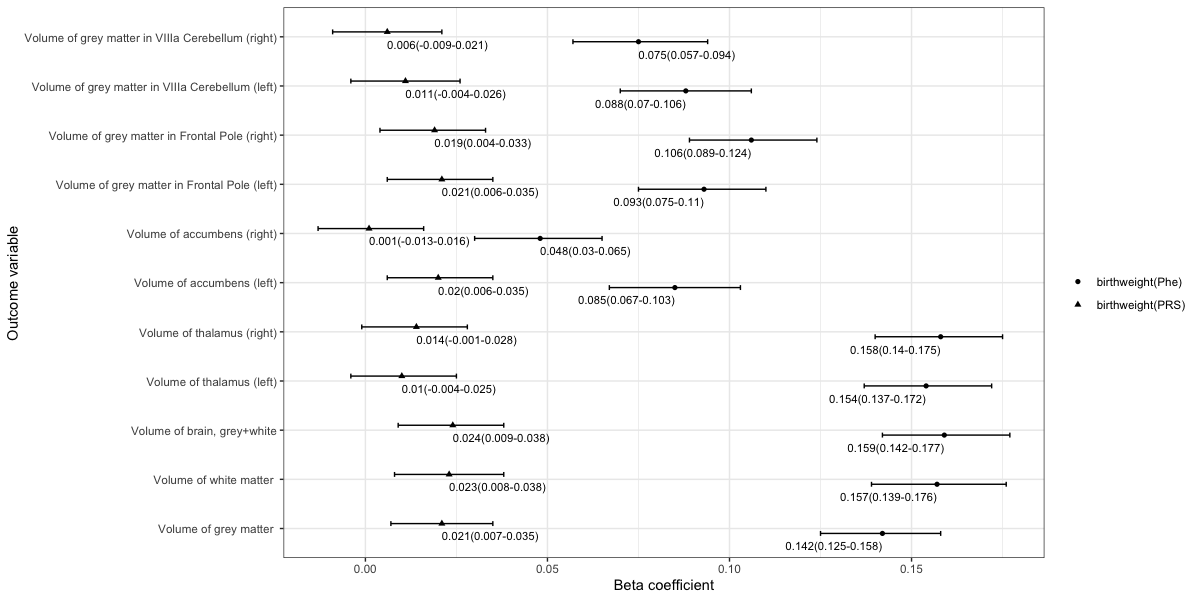


*The x-axis refers to beta coefficient. The y-axis represents the outcome variables. Points display the beta and 95% CIs (error bars) of beta coefficient. Birth weight PRS indicates the polygenic scores for birth weight. Birth weight Phenotype means the phenotype of birth weight. Detail information is showed in Table S2 in the Supplement.

**Figure S4. Associations between depression and brain volume for men**

**
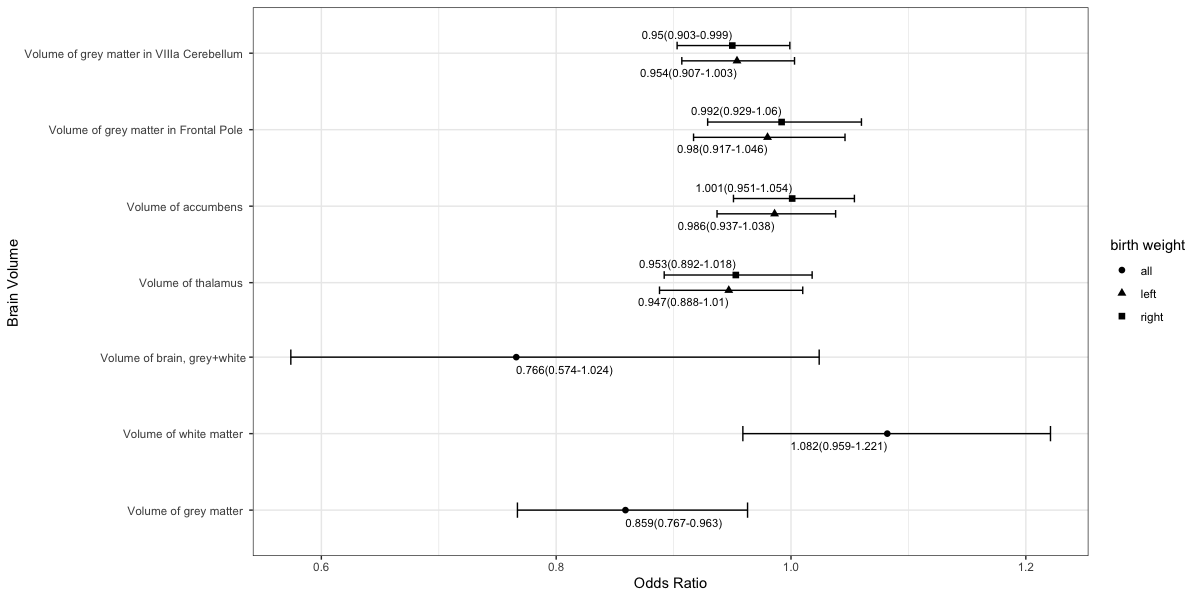
**

*The x-axis refers to odds ratio (OR). The y-axis represents the exposure variables. Points display the OR and 95% CIs (error bars) of OR. Detail information is showed in Table S3 in the Supplement.

**Figure S5. Associations between depression and brain volume for women**


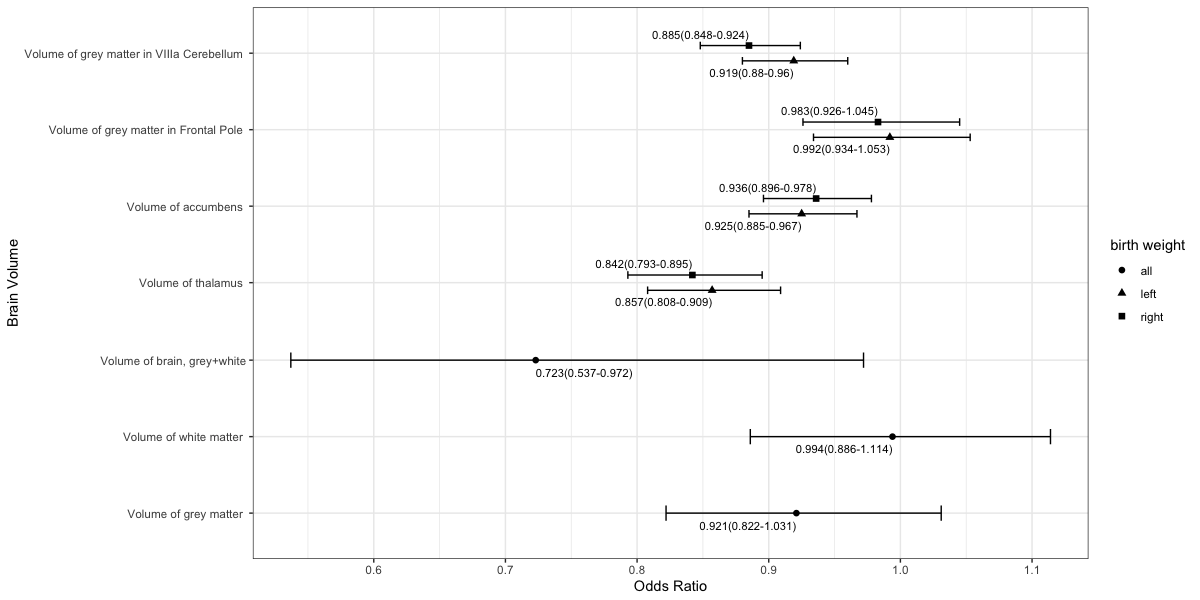


*The x-axis refers to odds ratio (OR). The y-axis represents the exposure variables. Points display the OR and 95% CIs (error bars) of OR. Detail information is showed in Table S3 in the Supplement.

**Figure S6. Association between birth weight and depression, via elevated levels of one of brain volumes for men, through steps approach**


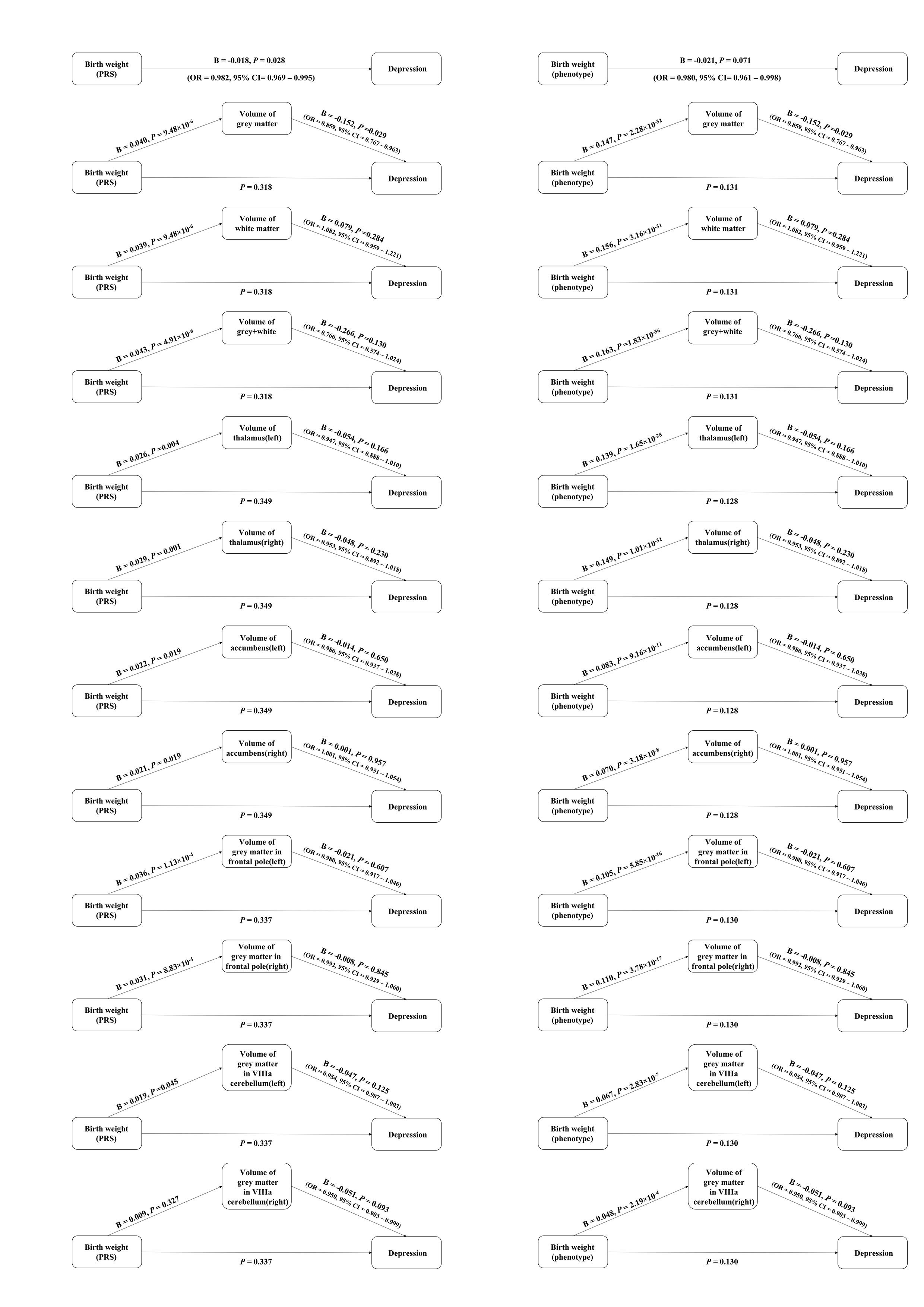


*Birth weight PRS indicates the polygenic scores for birth weight. Birth weight phenotype means the phenotype of birth weight.

**Figure S7. Association between birth weight and depression, via elevated levels of one of brain volumes for women, through steps approach**


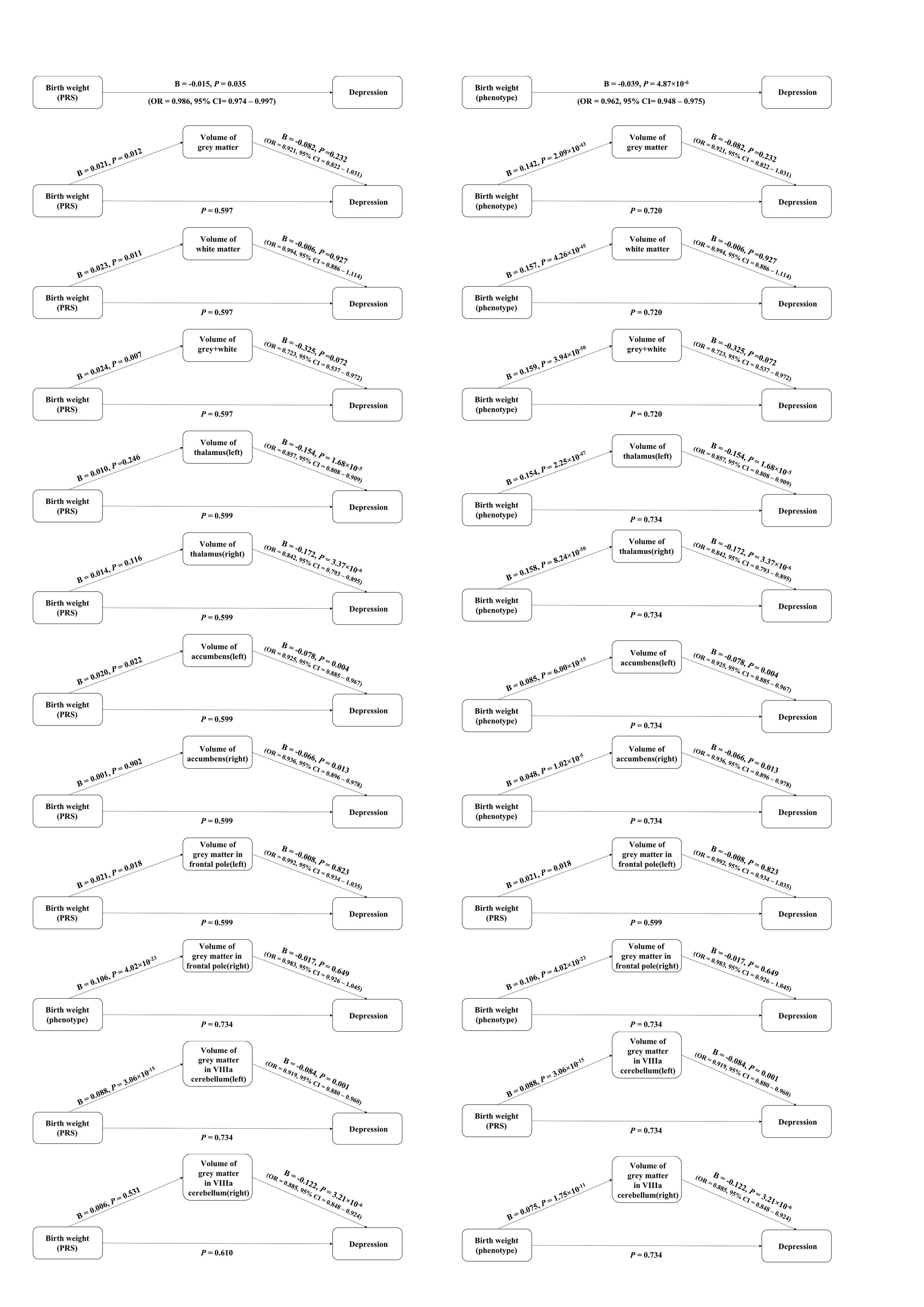


*Birth weight PRS indicates the polygenic scores for birth weight. Birth weight phenotype means the phenotype of birth weight.

Appendix：Questions of depression in UK Biobank

| Patient Health Questionnaire (PHQ-9) | | | | |  |
| --- | --- | --- | --- | --- | --- |
| 1. **20514** 2. **20510** 3. **20534** 4. **20519** 5. **20511** 6. **20507** 7. **20508** 8. **20518** 9. **20513** | Over the last 2 weeks, how often have you been bothered by any of the following problems?  a. Little interest or pleasure in doing things  b. Feeling down, depressed, or hopeless  c. Trouble falling or staying asleep, or  sleeping too much  d. Feeling tired or having little energy  e. Poor appetite or overeating  f. Feeling bad about yourself or that you  are a failure or have let yourself or your  family down  g. Trouble concentrating on things, such  as reading the newspaper or watching  television  h. Moving or speaking so slowly that other  people could have noticed? Or the opposite — being so fidgety or restless that you have been moving around a lot more than usual  i. Thoughts that you would be better off  dead or of hurting yourself in some way | | [Select one from the following for each of the statements]  - 01 Not at all  - 02 Several days  - 03 More than half the days  - 04 Nearly every day  - DA Prefer not to answer | |  |
| Core symptoms of depression based on CIDI | | | | | |
| **20446** | | Have you ever had a time in your life when you felt sad, blue, or depressed for two weeks or more in a row? | | [Select one from]  - 01 Yes  - 00 No  - DA Prefer not to answer | |
| **20441** | | Have you ever had a time in your life lasting two weeks or more when you lost interest in most things like hobbies, work, or activities that usually give you pleasure? | | [Select one from]  - 01 Yes  - 00 No  - DA Prefer not to answer | |

**Reference**

1. Davis, K.A.S. *et al.* Indicators of mental disorders in UK Biobank—A comparison of approaches. *International Journal of Methods in Psychiatric Research* **28**, e1796 (2019).

2. Kroenke, K., Spitzer, R.L., Williams, J.B.W. & L?we, B. The Patient Health Questionnaire Somatic, Anxiety, and Depressive Symptom Scales: a systematic review. **32**, 345-359.

3. Kessler, R.C., Andrews, G., Mroczek, D., Ustun, B. & Wittchen, H.l. The World Health Organization Composite International Diagnostic Interview short‐form (CIDI㏒F). *International Journal of Methods in Psychiatric Research* (1998).

4. Baron, R.M. & Kenny, D.A. The moderator-mediator variable distinction in social psychological research: conceptual, strategic, and statistical considerations. *J Pers Soc Psychol* **51**, 1173-82 (1986).

5. Bulmer & Martin. Charles M. Judd and David A. Kenny, Estimating the Effects of Social Interventions, Cambridge University Press, Cambridge, 1981. 243 pp. ￡19.50, paper ￡6.95. *Journal of Social Policy* **12**, 283 (1983).
